# Localization of CO_2_ Leakage from a Circular Hole on a Flat-Surface Structure Using a Circular Acoustic Emission Sensor Array

**DOI:** 10.3390/s16111951

**Published:** 2016-11-19

**Authors:** Xiwang Cui, Yong Yan, Miao Guo, Xiaojuan Han, Yonghui Hu

**Affiliations:** 1 School of Control and Computer Engineering, North China Electric Power University, Beijing 102206, China; y.yan@kent.ac.uk (Y.Y.); guomiao@ncepu.edu.cn (M.G.); hxj@ncepu.edu.cn (X.H.); yhhu@ncepu.edu.cn (Y.H.); 2 School of Engineering and Digital Arts, University of Kent, Canterbury, Kent CT2 7NT, UK

**Keywords:** CO_2_ leakage, leak localization, acoustic emission, circular sensor array

## Abstract

Leak localization is essential for the safety and maintenance of storage vessels. This study proposes a novel circular acoustic emission sensor array to realize the continuous CO_2_ leak localization from a circular hole on the surface of a large storage vessel in a carbon capture and storage system. Advantages of the proposed array are analyzed and compared with the common sparse arrays. Experiments were carried out on a laboratory-scale stainless steel plate and leak signals were obtained from a circular hole in the center of this flat-surface structure. In order to reduce the influence of the ambient noise and dispersion of the acoustic wave on the localization accuracy, ensemble empirical mode decomposition is deployed to extract the useful leak signal. The time differences between the signals from the adjacent sensors in the array are calculated through correlation signal processing before estimating the corresponding distance differences between the sensors. A hyperbolic positioning algorithm is used to identify the location of the circular leak hole. Results show that the circular sensor array has very good directivity toward the circular leak hole. Furthermore, an optimized method is proposed by changing the position of the circular sensor array on the flat-surface structure or adding another circular sensor array to identify the direction of the circular leak hole. Experiential results obtained on a 100 cm × 100 cm stainless steel plate demonstrate that the full-scale error in the leak localization is within 0.6%.

## 1. Introduction

Storage vessels and containers are widely used in a range of industries. For instance, in the carbon capture and storage (CCS) process, storage vessels are used to store and transport the captured CO_2_ [1]. Accidental leaks from CO_2_ storage vessels may compromise the stability and safety of the CCS system and cause serious environmental pollution and financial damage [2]. Therefore, it is crucial to identify and locate any accidental leak rapidly when it occurs. At present, CO_2_ transportation can be performed by several ways such as pipelines, ships, motor carriers, and railways [3]. The current research of CO_2_ leak detection in the transportation process has mainly focused on pipelines [4,5]. There has been very little reported research on the leak detection for CO_2_ storage vessels.

The practical storage vessel used in the CO_2_ transportation process is commonly a spherical or cylindrical tank, so it is, in fact, a three-dimensional structure. The shell of the vessel is very large, so if a local area of the shell is to be analyzed and studied for leakage detection, this area of interest can be treated as a flat-surface structure. The flat-surface structure belongs to a two-dimensional (2D) construction, which is different from the one-dimensional long pipelines [6]. Therefore, the leak detection methods including sensor arrangement and localization algorithms are different from those for long pipelines. The detection methods that may be considered for 2D leakage detection include tracer, concentration alarm, and infrared imaging, etc. [7]. The tracer and concentration alarm methods depend on the diffusion of CO_2_, so it is time-consuming. Infrared imaging is a potential method but it is not cost-effective. For most leak detection applications, many infrared cameras are needed in order to cover a large area from multiple directions. The acoustic emission (AE) method has advantages of rapid response, compact sensor structure, low capital cost [8] and, thus, has a high potential for the leak detection and localization of CO_2_ on a flat-surface structure. 

The AE method needs a number of sensors to obtain as much information as possible from the detection area. In previous studies, several sensor array configurations have been proposed for the localization of a damage source in a 2D space [9]. The damage source was usually generated by pencil-lead breaks, impact of a foreign object, fiber or metal breakage, matrix cracking [10]. Although these damage sources were not generated by leakage and the sensors used were not always of the AE type, the array configurations provide useful reference in this study.

It is well known that, at least three sensors are required to locate a damage source in a 2D structure. In the traditional damage localization method based on time-of-flight triangulation at multiple receiving points, the crossing point or zone is the location of the damage source [11]. For a regular square plate, a sensor array with four sensors arranged on the four corners is a common layout [12]. In practical applications, the crossing zone may be large and, thus, increase the localization error because the distance between the sensors is large. For a large flat-surface structure, the resolution of source localization will increase with the number of sensors used. Niri et al. [13] proposed a source localization model based on a sparse array with a set of piezoelectric sensors. The distance between sensors in the sparse array was large in order to cover the whole structure. 

It is worth noting that most sensor arrays for the localization of a damage source in a 2D space are sparse or loose types, and the damage source is burst-type [14]. For the burst-type signal, it can be separated in the time-domain waveforms because of sharp rising and descending edges. Thus, it is relative easy to determine the time difference between different sensors through threshold-based procedures, peak detection techniques and, more robustly, cross-correlation methods. However, the signal generated by leakage is a continuous-type and it cannot be separated in the time-domain waveforms because of the difficulty to identify the first wavefront. Therefore, the localization of the continuous-type leakage is more challenging than the burst-type damage source to some extent. There has been very little reported research on continuous leak localization on storage vessels based on the AE method [15,16], especially for CO_2_ leakage detection. This paper proposes a novel circular AE sensor array to realize the continuous CO_2_ leak localization from a flat-surface structure on a storage vessel. The localization performance of the proposed sensor array in combination with ensemble empirical mode decomposition (EEMD), cross-correlation and hyperbolic positioning algorithms are investigated in this study. The practical leak hole on a structure is of all shapes and sizes, but the circular hole is a typical and fundamental case, as long as the size of the hole is “regular” (circular, roundish, etc.) and significantly smaller than the size of the structure. 

## 2. Methodology

### 2.1. Sensing Arrangement

**Figure 1:**
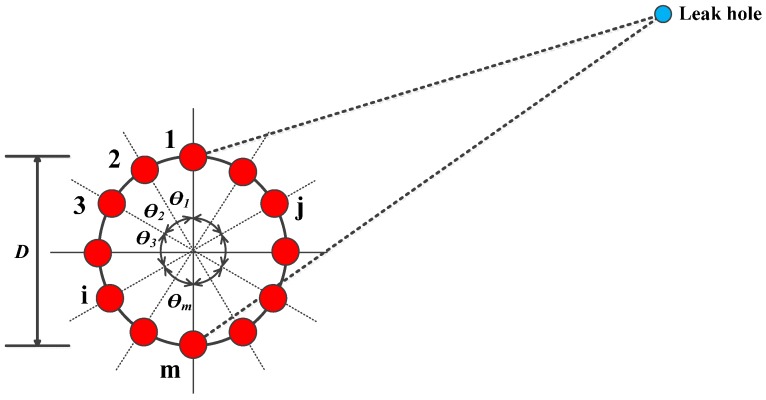
Circular sensor array arrangement with reference to the leak hole.

The key parameters of the circular array are the number of sensors, the angle between the adjacent sensors, and the diameter of the circle, as shown in Figure 1. In comparison with the sparse sensor arrays, the circular arrangement has advantages of compact layout, omnidirectional sensitivity, and similar signal attenuations and dispersions between the different sensors in the array, which are beneficial to correlation signal analysis. In addition, the distance difference between any two sensors in the array with reference to the leak hole is no greater than the diameter of the circle regardless of the location of the leak hole. Only when the leak hole on a diameter, such as the one connecting Sensors *i* and *j* in Figure 1, the distance difference between the two sensors is equal to the diameter of the circle. For all other cases, the distance difference is smaller than the diameter. Therefore, this restricted condition can be used as a threshold to assess the correlation results.

### 2.2. Leak Localization Principle

**Figure 2:**
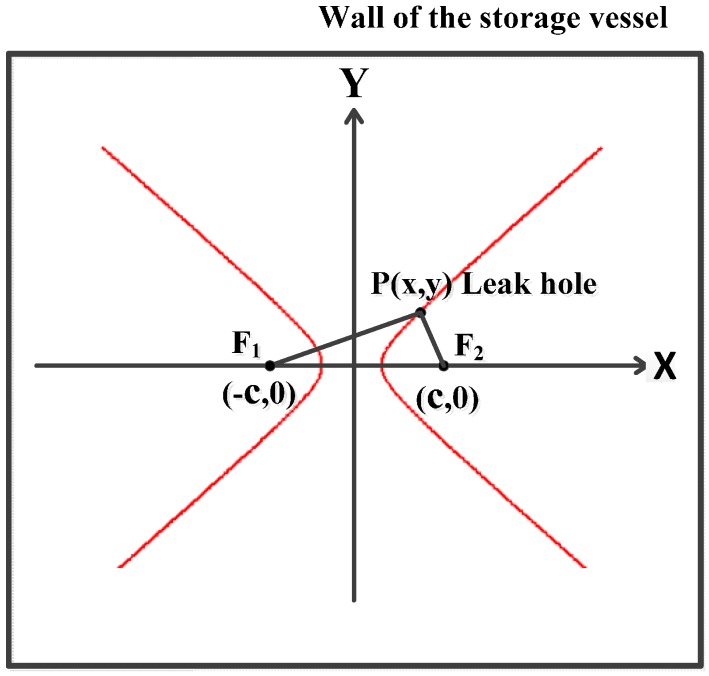
Principle of the localization.

Assume two sensors are installed on a plate, then a coordinate system can be established where the middle point of these two sensors is the origin, and the connecting line of these two sensors is the horizontal *x-*axis. Suppose the spacing between the two sensors is 2*c*, then the coordinates of Sensors 1 and 2 are (−*c*, 0) and (*c*, 0), respectively, as shown in Figure 2.

If the leak hole is located at the point *P*(*x*, *y*), the following equations can be derived based on the fundamental principle that a wave takes the minimum energy path to travel between two points [3]:

(1) $$PF_{1}=\sqrt{{\left(x+c\right)}^{2}+y^{2}}=vt_{1}$$

(2) $$PF_{2}=\sqrt{{\left(x-c\right)}^{2}+y^{2}}=vt_{2}$$

(3) $$|PF_{1}-PF_{2}|=v\Delta t$$

where *PF*_1_ and *PF*_2_ are the distances from the leak hole to the Sensors 1 and 2, respectively, *v* is the speed of AE wave, *t*_1_ and *t*_2_ are the arrival times of Sensors 1 and 2, and Δ*t* is the time difference between *t*_1_ and *t*_2_.

According to the geometrical relationship, the leak hole (point *P*) is on a hyperbolic curve when Equation (4) is satisfied. Therefore, the leak hole can be accurately located through the hyperbolic curves’ intersection when the number of sensors is more than three:

(4) $$F_{1}F_{2}>|PF_{1}-PF_{2}|>0$$

### 2.3. Ensemble Empirical Mode Decomposition

The original signal collected by the sensor array contains a lot of noise. Dispersion phenomenon will also make the signals produce distortion when the signals propagate along the flat-surface structure. Therefore, it is not accurate or sometimes even not feasible to locate the leak hole by directly cross-correlating the signals. In order to solve this problem, an appropriate denoising method or a mode decomposition algorithm should be applied. EEMD is an improved algorithm on the basis of the empirical mode decomposition (EMD) and overcomes some drawbacks of EMD such as modal mixing problem [17]. It is an effective approach to processing non-linear and non-stationary signals [18,19]. In comparison with other signal decomposition techniques, EEMD is an adaptive signal processing method which does not need prior information about the signal to be processed. In view of the non-linear and non-stationary characteristics of the leak signal, EEMD is a suitable method to decompose the signals in both time and frequency domains.

**Figure 3:**
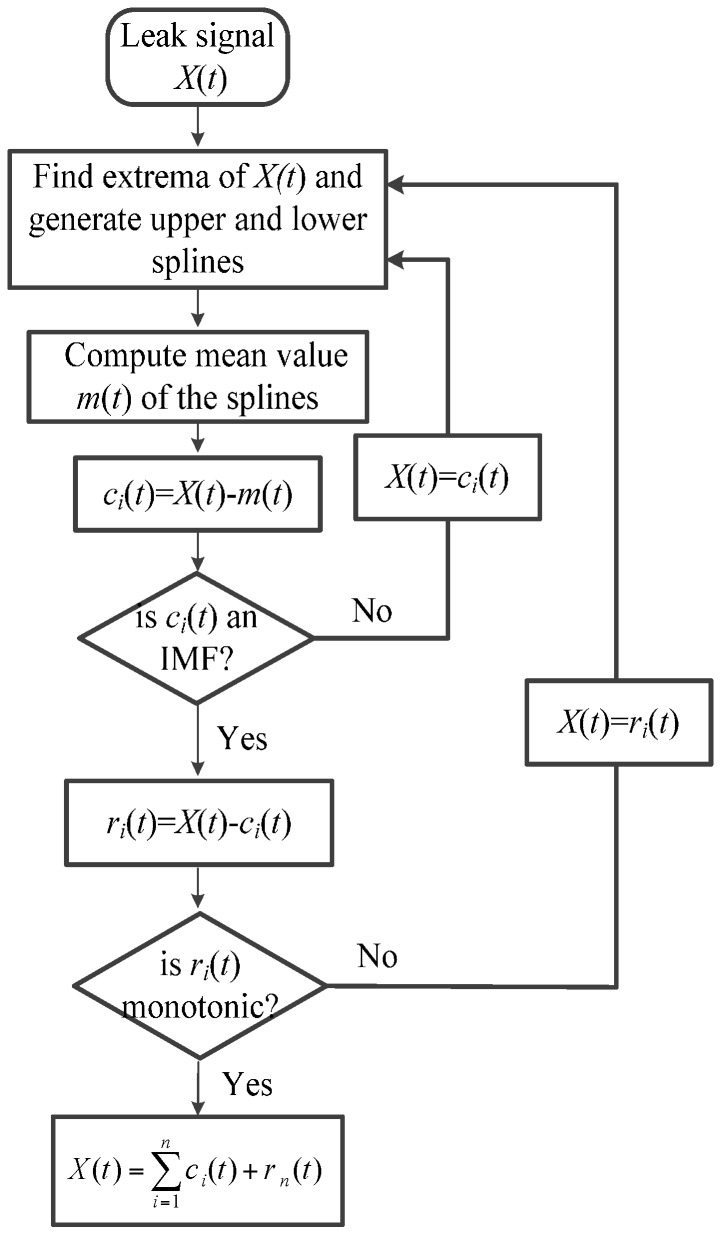
Flowchart of EMD.

EEMD is usually realized by decomposing the signal into a series of intrinsic mode functions (IMFs). The computational process of the EEMD is described as follows [20]:

(1) Add a Gaussian white noise signal *ω*(*t*) to the original signal *x*(*t*) to obtain a synthesized signal *X*(*t*).
  (5) X(t)=x(t)+ω(t)
(2) Decompose the synthesized signal using EMD into IMFs *c_i_*(*t*), as shown in Figure 3:
  (6) $$X(t)=\sum_{i=1}^{n}c_{i}(t)+r_{n}(t)$$

(3) Repeat Steps (1) and (2) *N* times, but add different white Gaussian noise each time:
  (7) $$X_{j}(t)=\sum_{i=1}^{n}c_{ji}(t)+r_{n}(t)$$
  The residue of added white noise should satisfy the following statistical rule [21]:
  (8) $$\varepsilon_{n}=\frac{\varepsilon }{\sqrt{N}}$$
  where *N* is the number of calculations, ε the root mean square (RMS) amplitude of the added noise, and *ε_n_* is the difference between the original data and the reconstructed data.
(4) Compute the ensemble means of corresponding IMFs as the final result:
  (9) $$c_{j}(t)=\frac{1}{N}\sum_{j=1}^{N}c_{ji}(t)$$

### 2.4. Cross-Correlation

The cross-correlation method is widely used for estimating the time delay in many research fields and has shown a very good performance. In this paper, the time difference is estimated through the following cross correlation computation [22].

(10) $$R_{xy}(m)=\frac{\sum_{k=0}^{N-\left|m\right|-1}x_{k}y_{k+m}}{\sqrt{\sum_{k=0}^{N-1}x_{k}^{2}}\sqrt{\sum_{k=0}^{N-1}y_{k}^{2}}}$$

where *x_k_* and *y_k_* denote the two leak signals from the two AE sensors and *N* is the length of the signal. The time difference corresponds to the location of the dominant peak in the correlation function *R_xy_*(*m*), whilst the peak value is the correlation coefficient representing the similarity of the two signals.

## 3. Experimental Results and Discussion

### 3.1. Experimental Setup

**Figure 4:**
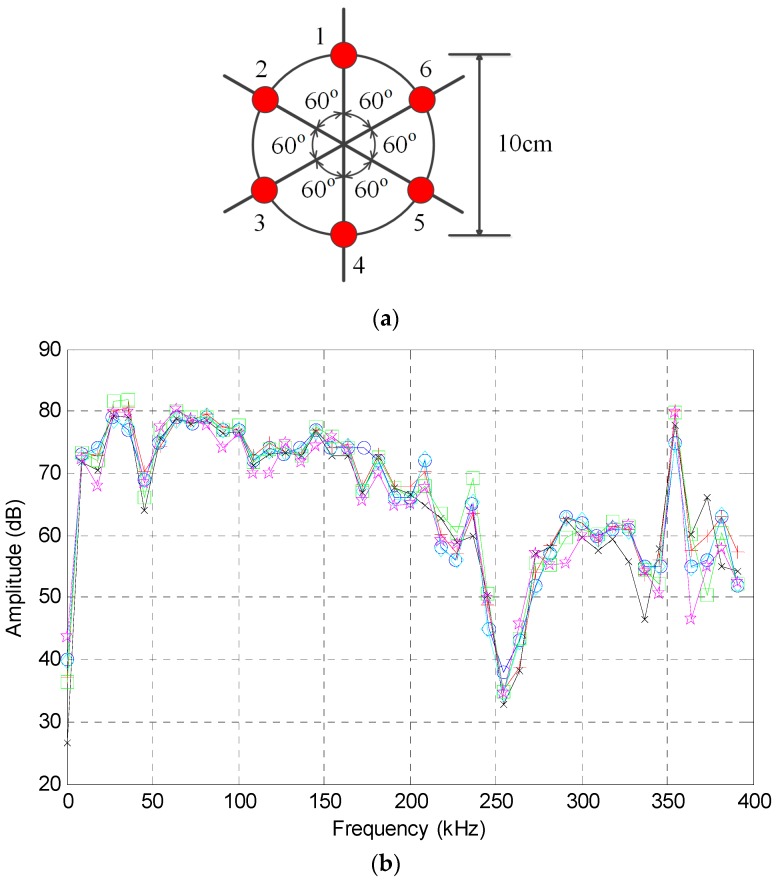
Sensor arrangement and the frequency response. (**a**) Sensor arrangement; and (**b**) frequency response of the AE sensor.

Laboratory-scale experiments were carried out on a 316L stainless plate with dimensions of 100 cm × 100 cm × 0.2 cm. A continuous leak of CO_2_ was created at a pressure of 2 bar from a hole of 2 mm in diameter in the center of the plate. An array with six identical high-frequency AE sensors (RS-2A, Softland Co., Ltd., Beijing, China) was mounted in a circular form on the plate using vacuum grease couplant. The angle between the adjacent sensors is 60° and the diameter of the circle is 10 cm. The sensor arrangement and the frequency response characteristics of all high-frequency AE sensors are shown in Figure 4. The consistency of the AE sensors is quite high, especially in the frequency band 150–200 kHz. The main technical specifications of the used AE sensors are shown in Table 1.

**Figure 5:**
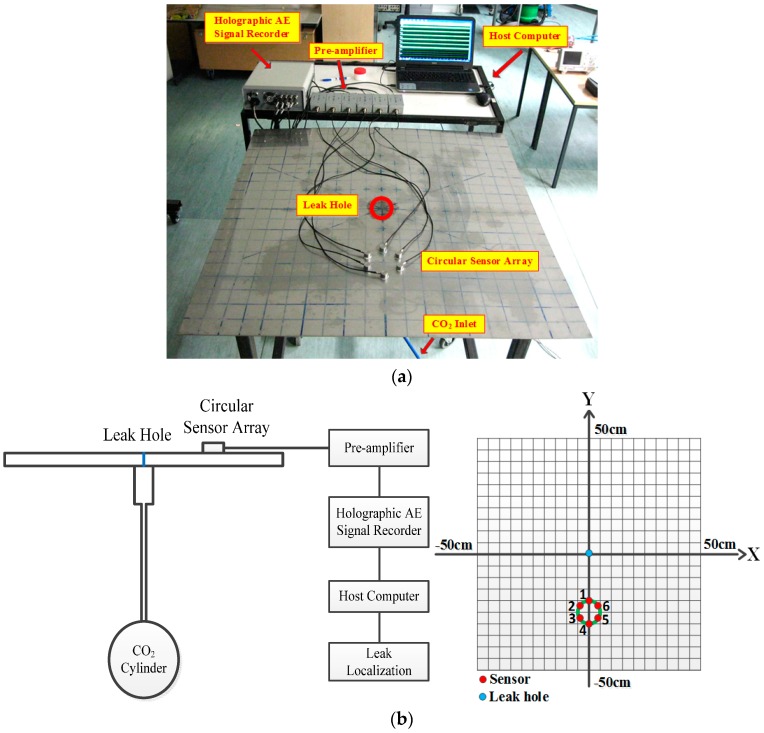
Experimental set-up and layout of the sensor array. (**a**) Experimental setup; and (**b**) the layout of the sensor array.

The acoustic signal were pre-amplified using AE amplifiers with a bandwidth of 10 kHz–1 MHz and a gain of 40 dB to boost the signal and reduce the effects of noise and interference. A holographic AE signal recorder (DS-8A, Softland Co., Ltd., Beijing, China) was used to acquire the waveforms at a sampling rate of 3 MHz. The A/D conversion resolution and input range of the signal recorder are 16 bits and ±10 V, respectively. The experimental set-up and sensor arrangement are shown in Figure 5.

### 3.2. Characteristics of the AE Leak Signal

**Figure 6:**
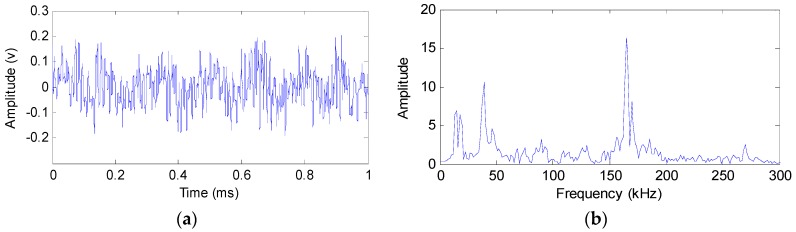
Time domain waveform and frequency spectrum of Sensor 1. (**a**) Time domain signal waveform; and (**b**) the frequency spectrum.

AE leak signals from the six AE sensors show very similar characteristics in view of the fact that they are mounted close to each other and are used to detect the same leak source. Take the signal from Sensor 1 (Figure 4a) as an example: the time domain waveform and corresponding frequency spectrum are plotted in Figure 6.

It can be seen from Figure 6 that the signal is continuous in the time domain and has a wide spectral range of 10–300 kHz. The signal contains frequency components in three main regions, with one in the high-frequency band (150–200 kHz) and the other two in the low-frequency band (10–50 kHz). Since the high-frequency region is not adversely affected by the common ambient noise, the signal in this region is utilized for the localization of the leak hole in this study.

**Figure 7:**
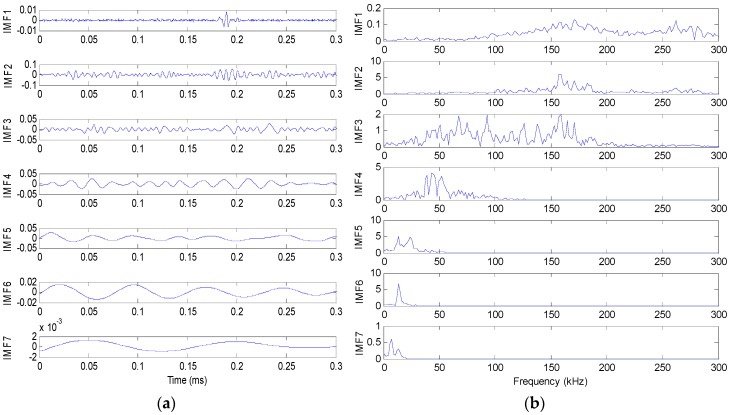
EEMD results of the leak signal. (**a**) Decomposed time domain signal waveforms; and (**b**) decomposed frequency spectra.

The original signal is decomposed using EEMD as discussed in Section 2. Figure 7 shows the EEMD decomposition results of the original signal from Sensor 1. Figure 7a is the decomposed time domain signal waveforms and Figure 7b is the corresponding frequency spectra. It can be seen that seven IMF components are generated. IMF1 has the highest frequency components while other IMF components contain lower frequency components. However, the energy of IMF1 is relatively low. Therefore, IMF2 is extracted to identify the location of the leak hole by comprehensively considering the frequency and energy of the signal.

### 3.3. Leak Localization Results and Error Analysis

The time difference between any pair of signals from the sensor array can be calculated through cross-correlation. The sensor array contains six sensing elements; therefore, there is a set of 15 cross-correlation results. If the speed of the AE signal is known, the distance difference can be calculated and then the leak hole located. The speed is found to be 4610 m/s, which was measured by conducting the Nielsen-Hsu pencil lead break test [23]. Table 2 shows the measured time difference and distance difference between the signal pairs. The implementation of leak localization consists of four key stages. In the first stage, EEMD is deployed to extract the useful signal from the noise. The characteristics of the leak signal are analyzed in both the time and frequency domains to select the proper frequency band. The second step is to estimate the time differences between the sensor signals through correlation signal processing. The time difference between any pair of the sensor signals is calculated in this step. The third stage estimates the distance difference between the sensing elements from the measured time differences and wave speed. It is worth noting that the distance difference must satisfy the restricted condition of the circular array and the hyperbolic curve as analyzed in Section 2. Finally, a hyperbolic positioning algorithm is used to locate the leak hole by finding the crossing points of the hyperbolic curves.

**Figure 8:**
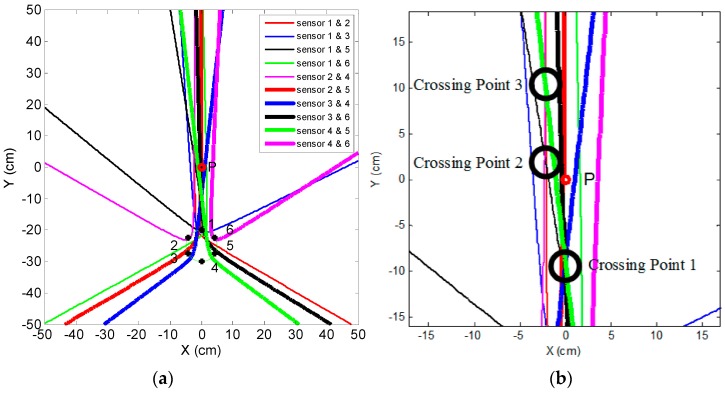
Leak localization result. (**a**) Leak localization plot; and (**b**) a zoomed-in version around the leak hole.

It can be seen from Table 2 that the absolute error in the determination of the distance difference is no greater than 0.6 cm. This result indicates good cross-correlation performance of the AE sensor array in a circle. In addition, the results from 1 and 4, 2 and 3, 2 and 6, 3 and 5, and 5 and 6 cannot satisfy the condition of the hyperbolic curve as analyzed in Section 2; therefore, there are a total of ten hyperbolic curves created. The leak localization results, arising from the hyperbolic positioning algorithm, are shown in Figure 8a. The crossing points of hyperbolic curves around the leak hole are seen in a zoomed-in version in Figure 8b.

In theory, all hyperbolic curves should intersect at one point (i.e., the leak hole); however, in practice there is more than one crossing point formed by two, three, or more curves due to errors in measurement, as shown in Figure 8a. It can be seen from Figure 8b that three crossing points formed by at least three curves around the leak hole and their coordinates are (−0.2 cm, −9.2 cm), (−2.2 cm, 2.6 cm), and (−2.2 cm, 10.2 cm), respectively. Among them, crossing Point 1 is formed by five curves while crossing Points 2 and 3 are formed by three curves, respectively. The rule to locate the leak hole is based on the fact that the crossing point has a higher probability to be the leak source if it formed by more curves. In this study, the location of the leak hole is, thus, estimated using the following equation.

(11) $$(x,y)=\frac{\sum_{i=1}^{m}(x_{i},y_{i})n_{i}}{\sum_{i=1}^{m}n_{i}}$$

where (*x_i_*, *y_i_*) is the coordinate of the *i*th crossing point, and *n_i_* is the number of the crossing curves of the *i*th crossing point. 

The resulting coordinates of the leak hole in this example are (−1.3 cm, −0.7 cm). The absolute error in this localization is no greater than 2 cm on the 100 cm × 100 cm plate. It must be noted that the time difference measurement is crucial in the whole localization process and even a small error can corrupt the localization result. The time difference calculated through cross-correlation usually contains several peak values. Errors will be introduced if the wrong peak is selected. In order to enhance the stability and accuracy of the localization, an optimized method is proposed by changing the position of the circular AE sensor array on the flat-surface structure or adding another circular sensor array to identify the direction of the leak hole.

**Figure 9:**
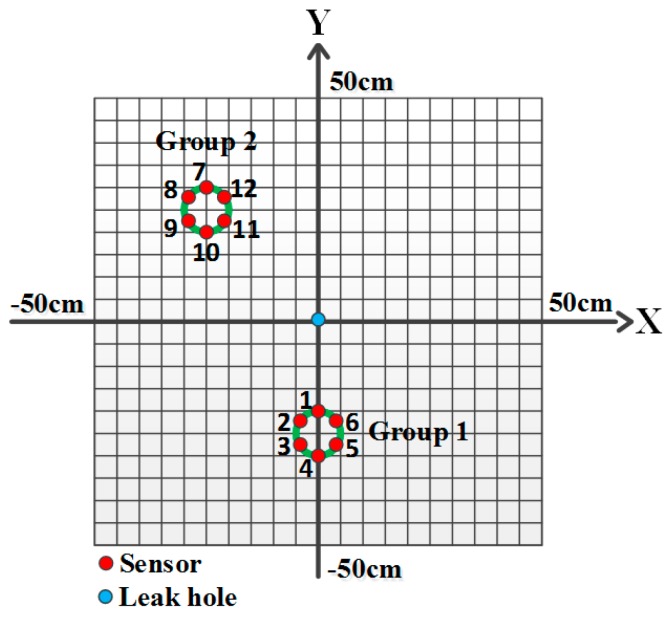
An optimized array arrangement.

**Figure 10:**
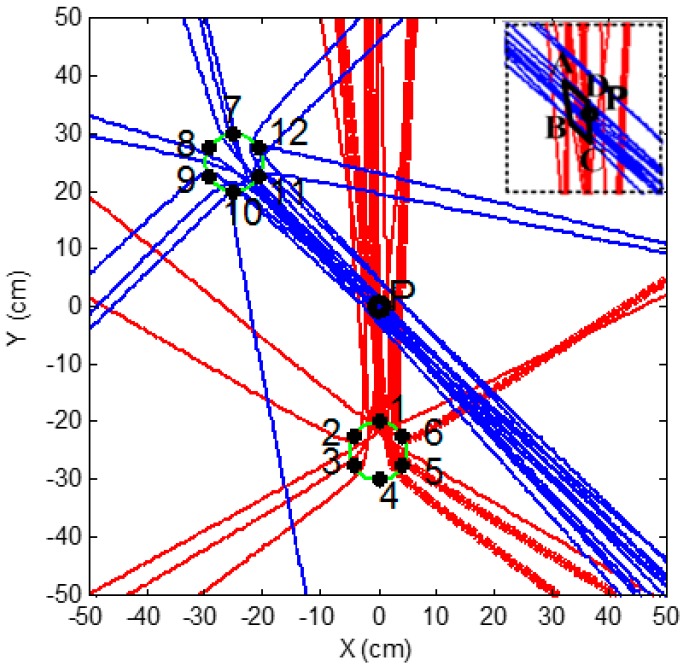
Localization results using the optimized method.

It can be seen from Figure 8a that intensive curves are toward the direction of the leak hole, although some curves do not pass through the leak hole. This phenomenon shows another advantage of the circular sensor array, i.e., it has a very good directivity. If changing the position of the sensor array or adding another array, a new direction will be toward to the leak hole. Thus, the leak hole can be located by the two directions. The optimized sensor arrangement is shown in Figure 9 and the localization results using this optimized method are shown in Figure 10.

**Figure 11:**
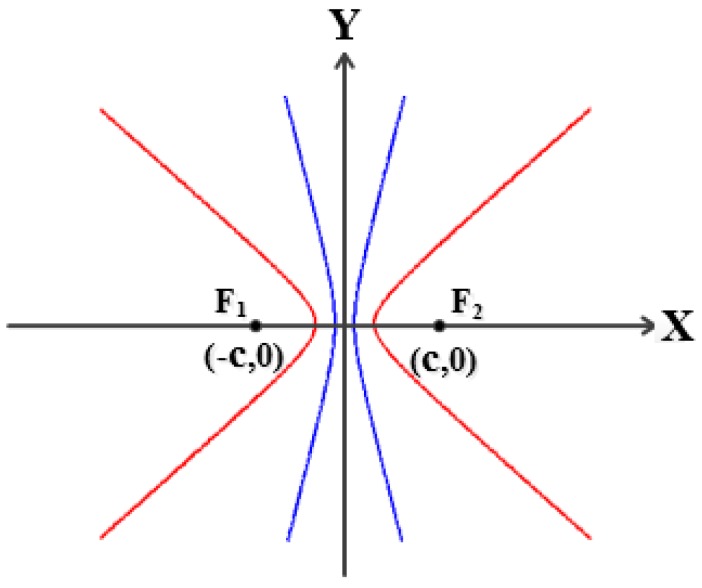
Diagram of the hyperbolic curves.

Figure 10 shows that both sensor arrays can find the direction of the leak hole, which is in the narrow crossing zone. This narrow crossing zone is shown more clearly in the upper right dashed box, a zoomed-in version. It can be seen from the zoomed-in version that the coordinates of four points of the crossing zone are A (−1.5 cm, 2.0 cm), B (−1.1 cm, −1.5 cm), C (0.2 cm, −2.2 cm) and D (0.1 cm, 1.1 cm), respectively. This result suggests that the leak hole can be located even when some hyperbolic curves deviate from the actual leak hole. Moreover, it can be seen that the directivity of Group 2 is better than that of Group 1. This is because that Group 2 is farther away from the leak hole, thus, the distance difference from any two sensors in the array with reference to leak hole (|*PF*_1_ − *PF*_2_|) is smaller. Therefore, the opening angle of the hyperbolic curve is greater and the curve is more like a straight line (blue line in Figure 11), and the directivity of the sensor array is better. The final localization results using the optimized method are (−0.6 cm, −0.1 cm) by calculating the average of coordinates of four points in the narrow crossing area. In summary, the absolute error is 1.5 cm and the full-scale error is 0.6% (the full-scale error is defined as the absolute error normalized to the full length of the square plate).

**Figure 12:**
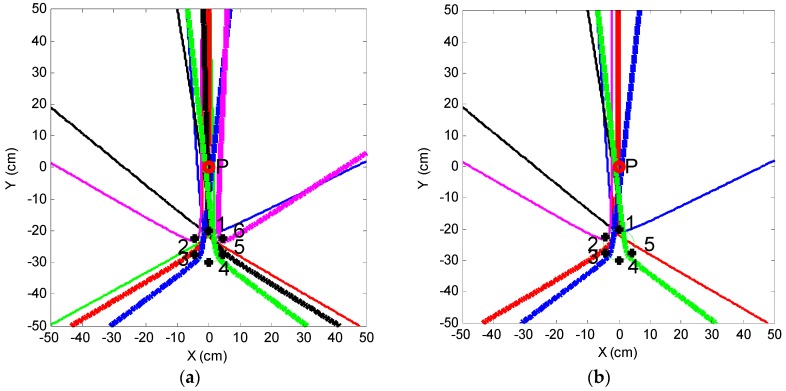

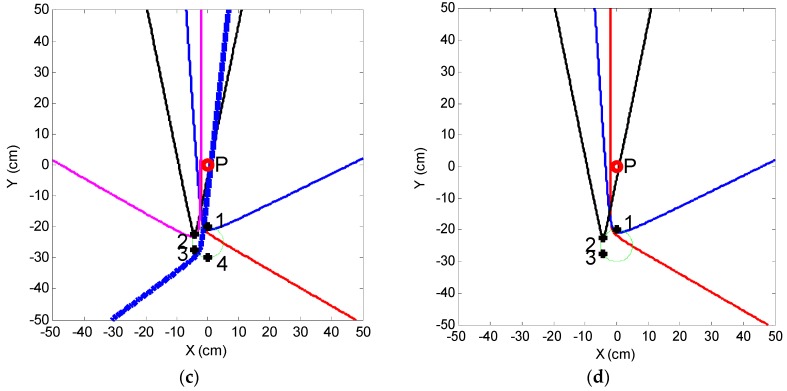
Comparison of the localization results. (**a**) Six sensors; (**b**) five sensors; (**c**) four sensors and (**d**) three sensors.

For a large detection area, the accuracy of leak localization will decrease when reducing the number of sensors used. In order to study the accuracy of the system with one or more faulty sensors in a practical application [24,25], the localization results of six, five, four and three sensors are compared, respectively, as shown in Figure 12.

It can be seen from Figure 12 that the localization errors with six, five, four and three sensors are 1.5 cm, 1.7 cm, 3.5 cm and 13 cm, respectively. These localization results and errors are calculated according to the process described in Section 3.3. 

Thus, the localization accuracy can satisfy requirements of most engineering applications when the number of sensors are more than four. In fact, the array will not be called a circular array and the localization method is not suitable if the number of sensors is less than four. It is believed that the proposed circular sensor array and localization method will show better performance if more sensors are used. However, this will require more computational and hardware costs.

## 4. Conclusions

In this study, a novel circular sensor array has been proposed to locate the CO_2_ leak hole on a flat-surface structure. Advantages of the proposed sensor array have been analyzed. The AE leak signals are decomposed into seven IMF components using EEMD and the signal component of IMF2 with high frequency and high energy has been used to predict the location of the leak hole through estimation of the time differences and distance differences of the sensor array. A total of ten hyperbolic curves are generated and intensive hyperbolic curves are toward the direction of the leak hole. There are three crossing points formed by at least three curves around the leak hole. A localization rule is defined based on the fact that the crossing point has a higher probability to be the leak source if it is formed by more curves. In order to enhance the stability and accuracy of the localization, an optimized method has been proposed by changing the position of the circular AE sensor array on the flat-surface structure or adding another circular sensor array to identify the direction of the leak hole. Experiential results demonstrate that the full-scale error in the leak localization is within 0.6% on a 100 cm × 100 cm stainless steel plate. Such an accuracy in leak localization should meet the requirement of most practical applications.

## Figures and Tables

**Table 1:** Technical specifications of the AE sensor.

Parameter	Specification
Material	Piezoelectric ceramic
Diameter	18.8 mm
Height	15 mm
Operating temperature	−20–200 °C
Operating frequency range	50–400 kHz

**Table 2:** Measured distance difference and corresponding error.

	Actual Distance between Two Sensors *F*_1_*F*_2_ (cm)	Measured Distance Difference |*PF*_1_ − *PF*_2_| (cm)	Actual Distance Difference (cm)	Absolute Error (cm)
1 & 2	5.0	2.5	2.9	−0.4
1 & 3	8.7	7.2	7.8	−0.6
1 & 4	10.0	10.5	10.0	0.5
1 & 5	8.7	8.1	7.8	0.3
1 & 6	5.0	2.6	2.9	−0.3
2 & 3	5.0	5.2	4.9	0.3
2 & 4	8.7	7.5	7.1	0.4
2 & 5	10.0	5.0	4.9	0.1
2 & 6	8.7	0.0	0.0	0.0
3 & 4	5.0	2.0	2.2	−0.2
3 & 5	8.7	0.0	0.0	0.0
3 & 6	10.0	4.8	4.9	−0.1
4 & 5	5.0	2.0	2.2	−0.2
4 & 6	8.7	7.7	7.1	0.6
5 & 6	5.0	5.2	4.9	0.3
